# Effects of gender in resident evaluations and certifying examination pass rates

**DOI:** 10.1186/s12909-018-1440-7

**Published:** 2019-01-07

**Authors:** Melanie S. Sulistio, Amit Khera, Kathryn Squiers, Monika Sanghavi, Colby R. Ayers, Weifeng Weng, Salahuddin Kazi, James de Lemos, David H. Johnson, Lynne Kirk

**Affiliations:** 1 Department of Internal Medicine, University of Texas Southwestern Medical Center, 5323 Harry Hines Blvd, Dallas, TX 75390-8830 USA; 2 Division of Cardiology, Dallas, USA; 3 Department of Clinical Sciences, Dallas, USA; 4 University of Texas Southwestern Medical Center, Dallas, TX USA; 5 American Board of Internal Medicine, Philadelphia, PA USA

**Keywords:** Gender, Resident evaluations, Certification exam, Medical education

## Abstract

**Background:**

Though the proportion of female Internal Medicine (IM) residents and faculty has increased, there is minimal large scale modern data comparing resident performance by gender. This study sought to examine the effects of resident and faculty gender on resident evaluations.

**Methods:**

Retrospective observational study over 5 years in a single IM program. IM certifying examination pass rates were obtained from the American Board of IM.

**Results:**

Four hundred eighty-eight residents (195 women, 293 men), evaluated by 430 attending physicians (163 women, 270 men) were included. Twelve thousand six hundred eighty-one evaluations between 2007 and 2012 were analyzed. Female residents scored higher in two domains (Medical Interviewing, and Interpersonal and Communication Skills) (*p* < 0.01 for each), with no significant difference between genders for the other domains (Medical Knowledge, Overall Patient Care, Physical Examination, Procedural Skills, Professionalism, Practice Based Learning and Improvement, System Based Practices and Overall score). There were no differences in scoring between female and male attending physicians. There were no differences in certifying examination scores between women and men among graduating residents. National pass rates for women were not statistically different to pass rates for men from 1987 to 2015.

**Conclusions:**

Data from one large academic medical center demonstrate higher ratings for female residents on performance domains reflecting bedside care and interpersonal skills, with similar scores for medical knowledge and remaining domains. No significant difference was seen locally in certifying examination scores, nor in recent national pass rates, an objective measure of medical knowledge. Despite imbalanced female representation in areas of medicine, our data suggest that gender-based disparities in Internal Medicine resident medical knowledge and physician competency are no longer present.

**Electronic supplementary material:**

The online version of this article (10.1186/s12909-018-1440-7) contains supplementary material, which is available to authorized users.

## Background

Historically, significant differences were observed in Internal Medicine (IM) graduate medical education evaluations and in certifying board examination scores according to gender of the resident physician. Program director ratings and evaluation scores were higher for men than women in areas such as medical knowledge, procedural skills and clinical judgment [1, 2]. Differences were also described according to attending physician gender, with male residents rated higher by male attending physicians in several domains [2]. While some contemporary data suggest that gender gaps in evaluation are narrowing [3–5], there continues to be evidence of gender bias [6–9], which may be a key factor in the disparity in female representation beyond residency, specifically in academic leadership [10, 11] and in the subspecialties of Cardiovascular disease, Critical Care Medicine and Pulmonary disease, and Gastroenterology [12].

Differences between male and female resident performance on the certifying examination in IM of the American Board of Internal Medicine (ABIM) have also narrowed over time. Previously published reports found between 1973 and 1987, men had higher pass rates than women [1, 13]. But over the period between 1989 and 1992 the gender gap in the ABIM initial certifying examination in Internal Medicine performance appeared to close [14]. However, no further studies have investigated whether this finding persisted or has changed over time.

Further investigation of gender based differences in evaluation of resident performance in the modern era is necessary to monitor for trends and evidence of change, as this may elucidate whether differences in resident performance and medical knowledge influence gender discrepancies beyond residency. Also, performance evaluations have evolved to include the core competencies of the Accreditation Council for Graduate Medical Education (ACGME) [15]. These core competency evaluations provide a more comprehensive assessment of the skills necessary for medical practice. Very few large scale, contemporary studies of the effects of resident and faculty gender on resident performance evaluations have been performed in IM. Thus, as a first step in determining the association of gender on performance assessment in internal medicine and its subspecialties, we aimed to describe contemporary gender-differences in evaluation ratings and ABIM certification examination results using local and national data.

## Methods

Subjects were 488 Internal Medicine residents and 430 faculty at the University of Texas (UT) Southwestern Medical Center between 2007 and 2012. Included residents were enrolled either in a 1 year Internal Medicine preliminary year program, or a 3 year Internal Medicine categorical residency program. Both categorical and preliminary residents were included so that findings regarding gender could be interpreted broadly across both groups. Internal Medicine faculty included those that supervised residents on inpatient clinical rotations. To maintain confidentiality, residents and faculty were assigned unique identifiers. The study was approved by the UT Southwestern Institutional Review Board.

Faculty evaluations of residents were completed using the online New Innovations Inc. (Uniontown, OH) platform, and maintained in a secure and confidential database. Outpatient, elective and Clinical Evaluation Exercise evaluations were removed due to differences in reporting structure from the standard inpatient evaluation. Inpatient evaluations were also used because they were the majority of the total evaluations per learner. The evaluation consisted of 9 questions from 6 domains, plus an overall rating score. The domains were defined as the ACGME Core Competencies and included Patient Care, Medical Knowledge, Practice Based Learning and Improvement (PBL), Interpersonal and Communication Skills, Professionalism and System Based Practice (SBP) [15] (see Additional file 1). Each domain received a single score except Patient Care, which was categorized into four subdomains, including Overall Patient Care, Medical Interviewing, Physical Examination, and Procedural Skills. Each category was measured via a Likert scale from 1 to 9 with an additional option of “No Interaction.”

First attempt scores from the ABIM initial certifying examination in Internal Medicine were obtained for residents who completed the 3 year IM Residency program. Residents who were enrolled in the 1 year IM preliminary program were excluded from analyses of examination scores.

Pass rates on the ABIM certifying examination for first time takers were examined from 1972 to 2015, and compared between men and women using Chi-square tests. 1972 was chosen as the initial year for analysis because this year demarcated the elimination of the oral portion of the examination.

### Statistical analysis

Mean scores (+/− standard deviation) for each domain and each resident were determined by averaging scores across all years of data available. Unpaired two-sample Student’s t-test was used to compare difference in evaluation scores across the domains for male versus female residents and male versus female faculty. Summary statistics for the equated score for female and male residents were determined by calculating the median and interquartile ranges. Significance was assessed via the Wilcoxon Rank-Sum test. Spearman rank correlation coefficients were calculated to determine the relationship between each resident’s average domain score with their equated score on the ABIM certifying examination. Because of the inherent yearly variation in the examination, the scores were equated based on item response theory. To account for multiple comparisons, a significance level of alpha = 0.01 was used. All analyses were performed using SAS software, version 9.4 (SAS Institute, Cary, NC, USA).

## Results

A total of 488 residents were included, 40% of whom were women. The number of faculty who completed evaluations was 430, of whom 38% were women. The total number of evaluations were 16,623. Three thousand nine hundred forty-two outpatient, elective and Clinical Evaluation Exercise evaluations were excluded from this total leaving 12,681 remaining to be analyzed. Of the residents included, 355 completed the categorical IM program and received a score for the initial ABIM certifying examination in IM.

Women and men received similar overall summary scores (7.27 vs 7.19, *p* = 0.12). Differences in ratings by gender were observed for Medical Interviewing and Interpersonal and Communication Skills where women were rated higher than men (Table 1). There was borderline significance in Overall Patient Care (*p* = 0.012). Women and men were rated similarly in Medical Knowledge, Physical Examination, Procedural Skills, Professionalism, Practice Based Learning, and System Based Practices (Table 1). There were no significant differences in evaluation results according to gender of the faculty (Table 2) (p-interaction > 0.10, each domain). Both male and female faculty tended to rate female residents higher in the aforementioned categories.

**Table 1:** Evaluation scores by domain stratified by gender of resident

Characteristic	Female Residents *n* = 195 (40)	Male Residents *n* = 293 (60)	*P* value
Overall	7.27 (1.09)	7.19 (1.13)	0.12
Medical Knowledge	7.21 (0.9)	7.22 (0.97)	0.51
Patient Care			
Patient Care, Overall	7.5 (0.94)	7.39 (1)	0.01
Medical Interviewing	7.41 (0.86)	7.30 (0.91)	0.004
Physical Examination	7.28 (0.86)	7.21 (0.89)	0.05
Procedural Skills	7.42 (0.84)	7.42 (0.87)	0.95
Professionalism	7.79 (0.89)	7.70 (0.94)	0.02
Practice Based Learning	7.41 (0.88)	7.36 (0.91)	0.11
Interpersonal and Communication Skills	7.61 (0.9)	7.47 (0.96)	0.001
System Based Practices	7.37 (0.86)	7.30 (0.9)	0.09

**Table 2:** Evaluations of female and male residents stratified by gender of faculty

Faculty Gender	Female *n* = 163 (38)			Male *n* = 270 (62)			P interaction
Resident Gender	Female *n* = 62	Male *n* = 101	*p*-value	Female *n* = 100	Male *n* = 170	*p*-value	
Overall	7.23 (0.6)	7.15 (0.67)	0.27	7.22 (0.51)	7.17 (0.55)	0.11	0.54
Medical Knowledge	7.18 (0.51)	7.18 (0.58)	0.82	7.17 (0.45)	7.21 (0.53)	0.44	0.24
Patient Care							
Patient Care, Overall	7.45 (0.58)	7.32 (0.58)	0.02	7.48 (0.47)	7.38 (0.52)	0.04	0.34
Medical Interviewing	7.40 (0.49)	7.28 (0.54)	0.009	7.37 (0.4)	7.28 (0.48)	0.02	0.29
Physical Examination	7.27 (0.49)	7.17 (0.53)	0.06	7.25 (0.37)	7.19 (0.46)	0.11	0.12
Procedural Skills	7.35 (0.61)	7.25 (0.68)	0.13	7.36 (0.49)	7.40 (0.52)	0.46	0.16
Professionalism	7.73 (0.51)	7.63 (0.54)	0.05	7.77 (0.38)	7.70 (0.41)	0.04	0.08
Practice Based Learning	7.40 (0.51)	7.32 (0.53)	0.10	7.39 (0.42)	7.35 (0.46)	0.16	0.70
Interpersonal and Communication Skills	7.60 (0.51)	7.47 (0.57)	0.01	7.59 (0.45)	7.45 (0.51)	0.001	0.62
System Based Practices	7.34 (0.5)	7.25 (0.54)	0.07	7.34 (0.4)	7.30 (0.46)	0.20	0.22

**Fig. 1:**
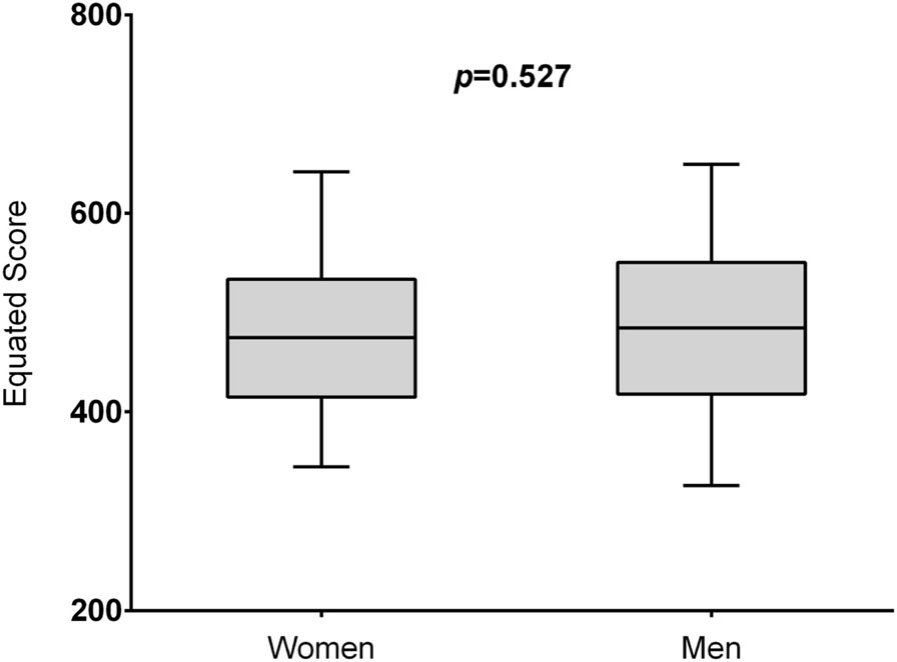
ABIM certification examination in Internal Medicine scores by gender of residents The vertical axis is the average equated score of the residents’ examinations by gender with indicated confidence intervals. The horizontal axis specifies the gender of the residents

No differences were observed in mean ABIM certifying examination scores between male and female graduates of the training program over the 5 year study period (487 ± 96 vs 481 ± 90, *p* = 0.53) (Fig. 1). Correlations between mean resident evaluation ratings and scores on the certifying examination demonstrated the highest correlations between the Medical Knowledge and Patient Care evaluation domains and the test results (Table 3).

**Table 3:** Correlations between individual domain ratings and certifying examination scores

Domains	Overall	
	rho	*p*-value
Overall	0.12	0.03
Medical Knowledge	0.25	<.0001
Patient Care		
Patient Care, Overall	0.12	0.001
Medical Interviewing	0.12	0.003
Physical Examination	0.12	0.003
Procedural Skills	0.12	0.04
Professionalism	0.14	0.01
Practice Based Learning	0.17	0.003
Interpersonal and Communication Skills	0.08	0.17
System Based Practices	0.17	0.003

**Fig. 2:**
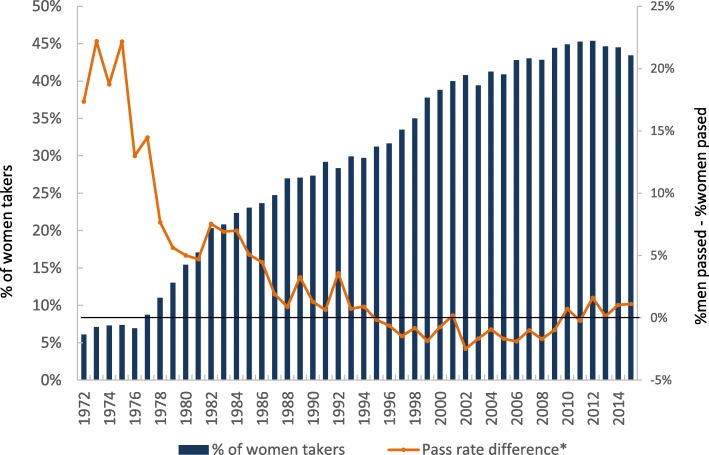
Gender gap in ABIM certification examination in Internal Medicine first time takers and pass rates This is a line graph with two vertical axes. The primary vertical axis is the percentage of women of the total number of first time takers. The secondary vertical axis is the difference between percent of men passing and percent of women passing for first time takers (i.e. men’s pass rate – women’s pass rate). The horizontal axis is the year of the certifying exam

Aggregate data on ABIM initial certifying examination in Internal Medicine performance for first time test takers since 1972 demonstrated a notable increase in the proportion of female examinees, from around 7% in years1972–1976, rising to 45% in the years 2010–2015 (Fig. 2). The pass rate of female examinees has increased steadily over time. Pass rates were initially 20% higher in male versus female examinees and this difference stayed statistically significant until 1986 (4.5% higher in male; corrected *P*-value = 0.045). Since year 1987, this difference is no longer statistically significant; ranging from male pass rate 3.6% higher (year 1992; corrected P-value = 0.26) to female pass rate 2.5% higher (year 2002; corrected P-value = 0.06) (Fig. 2).

## Discussion

In a large, academic Internal Medicine program, women now comprise approximately 40% of the resident workforce and of the teaching faculty. Our study has two principal findings: 1) female trainees are rated higher in two important aspects of clinical medicine by attending physicians in our institution, regardless of attending gender and 2) no significant difference is present in first time certifying examination pass rates between female and male trainees, either nationally or within the institution studied herein.

Despite the increase in women in medicine, the number of women in some subspecialties and in higher level academic positions continue to be sparse [10, 12]. Defining the reasons for this lack of parity, and the timeframe where attrition occurs is challenging. We evaluated one institution’s evaluations for gender differences to seek answers and spur discussion, and supplemented the data with certifying examination pass rates, both from that institution and nationally.

In our study, we found that female IM trainees were rated as having comparable levels of medical knowledge, and were rated higher in domains reflecting interpersonal skills by attending physicians. This is thought provoking, since it differs from another large study showing no difference in mean ratings by faculty evaluators regardless of faculty gender [5]. Women being rated higher in interpersonal domains, is consistent with previously published reports [16, 17]. This could be due to 1) a true difference in performance between women and men or (2) evaluator bias to expect women to have better, or men to have worse, skills in the interpersonal domains, or both. Because of the narrow scoring range, and the potential for evaluator implicit bias, we are compelled to exercise caution in overstating any conclusions. Certainly, this area deserves further attention and study.

It should be noted that evaluations analyzed were inpatient rotations, excluding outpatient rotations. As has been well documented in prior literature, the disproportionately higher amount of inpatient experience, as compared to outpatient experience [18], led us to this decision, but should be factored into its interpretation.

Given the subjectivity of the Likert scale evaluations, we sought corroborative data by obtaining ABIM initial certifying examination scores of the graduating residents. Not only did both the evaluations and the certifying examination scores show no significant difference between women and men, the most powerful correlation between certifying examination scores and a single competency was for Medical Knowledge, supporting the validity of the evaluations.

To further support our data, and to examine whether our data are generalizable to other institutions, we obtained national ABIM certifying examination in IM first attempt pass rates. Pass rates were used instead of score, due to known variability in the scoring and examination from year to year. In concordance with historical reports [14], men had higher pass rates from the 1970s until the 1980s. However, by 1987, there was no significant difference between women and men’s certification examination pass rates. The reason for the discrepancy in pass rates historically is beyond the scope of this study, but prior data supports that neither the method of scoring, nor changes in the types of medical schools or residency programs that the test-takers attended played a factor [13]. This is the first report that confirms that the gender gap in ABIM initial certifying examination pass rate remains closed in the modern era. The consistency of the local and national ABIM data supports the generalizability of our findings.

Despite our findings that suggest that perceived gender-based disparities in medical knowledge and physician competency no longer occur at the resident level, at least in our institution, women continue to be underrepresented in certain subspecialty fellowships and higher level academic positions [10, 12]. There are many contributing reasons [19–23]. As one example, a recent study found that male residents received more consistent and positive feedback as opposed to female residents [8], suggesting that gender bias is still pervasive in the medical education culture. Despite the widespread movement toward gender equality in the past 50 years [24], increased numbers of women in undergraduate medical education and graduate medical training [25, 26], and our data showing equivalent medical knowledge and women having better performance in patient care domains, there remains evidence of gender bias. Currently, close to half of all medical degrees are awarded to women [27]. Efforts to limit gender bias in career development, and to increase the number of female faculty in leadership positions to offer more opportunities for mentorship, coaching and role-modeling should be made to help achieve parity.

Whereas previously, male faculty tended to rate male residents higher [2], our data report demonstrates at one academic institution, there were no significant difference in ratings between female and male faculty. Additionally, women were rated higher in two domains. We speculate that the lack of women in leadership positions and in some subspecialty fellowships are not attributed to a difference in performance or knowledge with respect to gender.

## Limitations

The most important limitation to our data is that it was obtained from a single institution. However, it should be noted that this study has one of the largest number of evaluations, residents and faculty of any study of gender differences in IM graduate medical training to date [2, 3, 5, 28]. Moreover, the ratio of female residents to male residents in this study is comparable to national survey data [12].

Another limitation is that our survey instrument, while it was designed using American Board of Internal Medicine and American College of Graduate Medical Education recommendations for assessment, was a semi-quantitative Likert scale whose initial creation lacked traditional testing. While not a validated evaluation tool, the finding that medical knowledge had its highest correlation with ABIM certification exam pass rates suggests that our tool was effective. In addition, our tool can be viewed in Additional file 1 and be compared to evaluation instruments used at other institutions to draw conclusions regarding comparability. Our evaluations also demonstrated range restriction, with the vast majority of responses being between 5 and 8 on the Likert scale. This has been reported previously in the literature, and while a limitation, since commonly found, suggests that our findings may be similar to other institutions [29].

Our study was also limited by the inability to adjust for confounding factors. Learner demographics and data such as prior United States Medical Licensing Examination (USMLE) scores and in-training exam scores, as well as the resident evaluations of educators, peer and three-sixty evaluations, were unavailable at time of analysis. Our data was obtained prior to the conversion to milestone ratings by the ACGME in 2014. However, a recent study showed correlation of historical evaluation items to milestone ratings, as well as good correlation of both rating systems to certifying exam scores [30].

## Conclusion

This study is the one of the largest studies comparing resident performance in Internal Medicine between female and male residents. Female residents were rated higher in Medical Interviewing and in Interpersonal and Communication Skills, with similar ratings in the remaining domains. Men and women had similar performance on the initial ABIM certifying examination, both locally and nationally. These data suggest that gender differences in academic leadership positions and certain subspecialties such as cardiology cannot be attributed to gender differences in Internal Medicine resident medical knowledge and physician competency.

## Additional file

Additional file 1: Faculty Evaluation of Resident. (DOCX 17 kb)

## Abbreviations

ABIM: American Board of Internal Medicine
ACGME: Accreditation Council for Graduate Medical Education
IM: Internal Medicine
PBL: Practice Based Learning and Improvement
SBP: Systems Based Practice
USMLE: United States Medical Licensing Examination
UT: University of Texas
