# Supplementary material for: Effects of gender in resident evaluations and certifying examination pass rates
Source: BMC Med Educ. 2019 Jan 7;19:10. doi: 10.1186/s12909-018-1440-7 (PMC6322320; doi:10.1186/s12909-018-1440-7)
Supplement: Supplementary file 1 — Faculty Evaluation of Resident. (DOCX 17 kb) [file 12909_2018_1440_MOESM1_ESM.docx]

Additional file 1: Faculty Evaluation of Resident

1. Medical Knowledge

| Limited knowledge of basic and clinical sciences; minimal interest in learning; does not understand complex relations; mechanisms of disease | | | | | Exceptional knowledge of basic and clinical sciences, highly resourceful development of knowledge; comprehensive understanding of complex relationships, mechanisms of disease | | | | |  |
| --- | --- | --- | --- | --- | --- | --- | --- | --- | --- | --- |
| 1 | 2 | 3 | 4 | 5 | | 6 | 7 | 8 | 9 | No Interaction |

2a. Patient Care

| Often fails to discern relationship of medical facts and clinical data, evaluate alternatives, or consider risks and benefits. Is unaware of limitations of knowledge or skills. Frequently uses diagnostic procedures or therapies inappropriately. Indecisive in difficult management situations. Treats problems rather than patients. Does not consider patient preferences when making medical decisions. | | | | | Regularly integrates medical facts and clinical data, weighs alternatives, understands limitations of knowledge, and incorporates consideration of costs, risks and benefits. Wise use of diagnostic and therapeutic procedures. Reasons well in ambiguous situations. Spends time appropriate to the complexity of the problem. Superb review of data; always makes decisions based on available evidence, sound judgement, and patient preferences. | | | | |  |
| --- | --- | --- | --- | --- | --- | --- | --- | --- | --- | --- |
| 1 | 2 | 3 | 4 | 5 | | 6 | 7 | 8 | 9 | No Interaction |

2b. Medical Interviewing

| Incomplete, illogical, superficial, and not directed to patient’s problems. | | | | | Always precise, logical, thorough, reliable, purposeful, and efficient. Establishes a broad base of information about the patient. | | | | |  |
| --- | --- | --- | --- | --- | --- | --- | --- | --- | --- | --- |
| 1 | 2 | 3 | 4 | 5 | | 6 | 7 | 8 | 9 | No Interaction |

2c. Physical Examination

| Incomplete, inaccurate, cursory, non-directed, unreliable, and not directed to patient’s problems. | | | | | Always precise, logical, thorough, reliable, purposeful, and efficient. Establishes a broad base of information about the patient. | | | | |  |
| --- | --- | --- | --- | --- | --- | --- | --- | --- | --- | --- |
| 1 | 2 | 3 | 4 | 5 | | 6 | 7 | 8 | 9 | No Interaction |

2d. Procedural Skills

| Inept, careless. Frequent disregard for risk to patient and patient’s anxiety and comfort. | | | | | Always proficient and careful. Minimizes risk and discomfort to patients. Provides proper explanation of the purpose to the procedure. | | | | |  |
| --- | --- | --- | --- | --- | --- | --- | --- | --- | --- | --- |
| 1 | 2 | 3 | 4 | 5 | | 6 | 7 | 8 | 9 | No Interaction |

3. Professionalism

| Lacks respect, compassion, integrity, honesty; disregards need for self-assessment, fails to acknowledge errors; does not consider needs of patients, families or colleagues; does not display responsible behavior. | | | | | Always demonstrates respect, compassion, integrity, honesty; teaches/role models responsible behavior; total commitment to self-assessment; willingly acknowledge errors; consistently considers needs of patients, families, and colleagues. | | | | |  |
| --- | --- | --- | --- | --- | --- | --- | --- | --- | --- | --- |
| 1 | 2 | 3 | 4 | 5 | | 6 | 7 | 8 | 9 | No Interaction |

4. Practice-Based Learning and Improvement

| Fails to perform self-evaluation; lacks insight; resists or ignores feedback; fails to use information technology to enhance patient care or pursue self-improvement. | | | | | Constantly evaluates own performance. Incorporates feedback into improvement activities; effectively uses technology to manage information for patient care and self-improvement. | | | | |  |
| --- | --- | --- | --- | --- | --- | --- | --- | --- | --- | --- |
| 1 | 2 | 3 | 4 | 5 | | 6 | 7 | 8 | 9 | No Interaction |

5. Interpersonal and Communication Skills

| Does not establish even minimally effective therapeutic relationships with patients and families; does not demonstrate ability to build relationships through listening, narrative or nonverbal skills; does not provide education or counseling to patients, families or colleagues. | | | | | Establishes a highly effective therapeutic relationship with patients and families; demonstrates excellent relationship building through listening, narrative and nonverbal skills; excellent education and counseling of patients, families and colleagues; always “interpersonally” engaged. | | | | |  |
| --- | --- | --- | --- | --- | --- | --- | --- | --- | --- | --- |
| 1 | 2 | 3 | 4 | 5 | | 6 | 7 | 8 | 9 | No Interaction |

6. System-Based Practices

| Unable to access/mobilize outside resources; actively resists efforts to improve systems of care; does not use systematic approaches to reduce error and improve patient care. | | | | | Effectively accesses/utilizes outside resources; effectively uses systematic approaches to reduce errors and improve patient care; enthusiastically assists in developing systems improvement. | | | | |  |
| --- | --- | --- | --- | --- | --- | --- | --- | --- | --- | --- |
| 1 | 2 | 3 | 4 | 5 | | 6 | 7 | 8 | 9 | No Interaction |

7. Overall

| Unacceptable performance, should not proceed to the next level of training. | | Below average resident compared to those worked with in the past. | | | Average resident compared to those worked with in the past. | | | Above average resident compared to those worked with in the past. | | | Among the top residents, exceptional resident. | |
| --- | --- | --- | --- | --- | --- | --- | --- | --- | --- | --- | --- | --- |
| 1 | 2 | | 3 | 4 | | 5 | 6 | | 7 | 8 | | 9 |
